# Lower hypoglycemic but higher antiatherogenic effects of bitter melon than glibenclamide in type 2 diabetic patients

**DOI:** 10.1186/1475-2891-14-13

**Published:** 2015-01-26

**Authors:** Inayat U Rahman, Rooh Ullah Khan, Khalil Ur Rahman, Mohammad Bashir

**Affiliations:** Gandhara College of Pharmacy, Gandhara University, Peshawar, 25000 KPK Pakistan; Department of Physiology, Bannu Medical College, Bannu, KP Pakistan; Department of Anatomy, Bannu Medical College, Bannu, KP Pakistan; Department of Physiology, KIMS, Kohat, KP Pakistan

**Keywords:** Bitter melon, Glibenclamide, Type 2 diabetics, Glycemic control, Cardiovascular risk factors

## Abstract

**Objective:**

Since antiquity bitter melon has been in use for treating diabetes but clinical trials show conflicting results about its usefulness. The present study aims to asses and compare the hypoglycemic and antiatherogenic effects as well as the safety of two different doses of bitter melon with glibenclamide.

**Methods:**

A total of 95 participants were randomized into 3 groups; group I and group II received bitter melon (2 g/day and 4 g/day respectively) and group III received glibenclamide (5 mg/day) for 10 weeks. Glycemic control and antiatherogenic effects were determined by assessing glycohemoglobin (HbA1-c), fasting plasma glucose (FPG), 2 hour oral glucose tolerance test (OGTT), plasma sialic acid (PSA), systolic blood pressure (SBP), blood lipids and atherogenic index at different time periods.

**Results:**

Compared to baseline, mean reduction in HbA1-c at the endpoint was significant among patients of group I, group II and group III (p ≤ 0.05, *p* ≤ 0.02 and *p* < 0.005 respectively) and same was the case for FPG (*p* ≤ 0.05, p < 0.04, *p* < 0.003 respectively), but the improvement in 2 hour OGTT was significant only in group III (*p* < 0.03). The decrease in PSA was observed only among group I and group II with the later showing significant reduction from baseline (*p* < 0.01). In group III, the level slightly increased. Parameters including blood lipids, atherogenic index, body weight and SBP improved among patients of group I and group II but deteriorated among group III patients.

**Conclusions:**

Our study concludes that bitter melon has a weaker hypoglycemic effect but ameliorates the diabetes associated cardiovascular (CV) risk factors more effectively than glibenclamide.

**Trial registration:**

The trial was registered with Naseer Teaching Hospital Clinical Trials Registry number GU2014492233.

## Introduction

In developing world, herbal remedies are gaining popularity and 80-85% of their population relies upon alternative system of medicine. More than 7000 medicinal plants are currently in use by different cultures of the world to treat various conditions and almost 800 plants in the Asian subcontinent are shown to possess hypoglycemic activity [1, 2]. Among the various medicinal plants, bitter melon (family: Cucurbitaceae, genus: Momordica, species: Charantia) is the most popular and commonly used herb for treating diabetes [3]. There is uncertainty about the exact location of its origin, but it is cultivated in many tropical regions of India, Pakistan, China as well as South America and East Africa. Although, all parts of the plant have been shown to possess antidiabetic activities, the most commonly used part is the fruit pulp which is used in the form of juice or dried powder [4, 5]. A cluster of animal studies have documented the hypoglycemic effects of bitter melon fruit extracts [6–9]. Studies also indicate that glucose lowering effects of bitter melon fruit extract are comparable to some oral hypoglycemic agents used in the initial management of diabetes [10, 11]. Bitter melon is believed to exert its hypoglycemic effect through multiple mechanisms involving the stimulation or inhibition of the key enzymes of metabolic pathways. For example, it stimulates the key enzymes of hexose monophosphate pathway [12], increases the utilization of peripheral and skeletal muscle glucose [6, 7], prevents glucose uptake by intestine [13–15], inhibits gluconeogenesis [12, 16] and adipocytes differentiation [17] and normalizes the islet β cells [18, 19].

As compared to animal studies, only few clinical trials have been conducted so far regarding the hypoglycemic potential of bitter melon and majority of these studies lack proper control and have conflicting results [20–22]. Indeed, Diabetes United Kingdom (UK) has warned the people regarding the use of bitter melon capsules because of lack of information about its safety and recommended dose [23]. Because of the frequent use of processed bitter melon in the treatment of diabetes mellitus and the lack of information about its efficacy and safety, there is an urgent need to conduct a clinical trial comparing the hypoglycemic and/or antiatherogenic potential of bitter melon with oral hypoglycemic agent (OHA) and the present study is an attempt to evaluate and compare the antidiabetic and antiatherogenic effects as well as the safety of two different doses of bitter melon with glibenclamide in type 2 diabetic patients.

## Methods

A total of 112 type 2 diabetic patients, attending the medical ward of Naseer Teaching Hospital, Gandhara University, Peshawar, KPK, Pakistan, who had been previously managed with diet/exercise only for recently diagnosed type 2 diabetes (≤2 years) from the study screening were enrolled into the study and 95 were assigned to one of the 3 treatment groups (Table 1). Recruitment for the study was conducted according to the declaration of Helsinki (1997). The study protocol was approved by Gandhara University medical ethics committee. The volunteer patients of type 2 diabetes were explained the research protocol and their written informed consents obtained for the study. Participants were informed of their right to withdraw from the study at any time. They were advised not to take any drug without prior permission from investigator. Patients were screened for eligibility for study entry according to the criteria presented in below.

**Table 1:** **Baseline characteristics of type 2 diabetic patients**

Parameters	Bitter melon 2 g/day	Bitter melon 4 g/day	Glibenclamide (5 mg/day)
	(Group I)	(Group II)	(Group III)
N	30	31	29
Sex, M/F	21/9 (70/30)	20/11 (65/35)	18/11 (62/38)
Age (y)	51.90±10.50	52±11.40	52.20±8.70
Time since diagnosis of diabetes (yr)			
0-1	22 (73)	24 (77)	20 (70)
≤2	8 (27)	7 (23)	9 (30)
BMI (Kg/m^2^)	25.2±1.70	25.7±2.40	25±2.20

### Eligibility criteria for participation in the study

#### Inclusion criteria

- Type 2 diabetes mellitus (M/F) as diagnosed according to World health Organization 1999 (WHO) criteria
- FPG 126-240 mg/dl
- Age 30-70 years
- Normal Renal Function Tests
- Normal liver Function Tests

#### Exclusion criteria

- Previous use of antidiabetic drugs
- Presence of cancer
- Menopausal status or pregnancy
- Use of contraceptives
- Use of anticancer drugs
- Hormonal therapy
- Diabetic complications

### Study design

The present study is randomized, double blind, parallel group trial consisted of 2 weeks run-in period during which the patients received placebo and included reinforcement of lifestyle education to obtain fasting plasma glucose level between 126-240 mg/dl (7-13 mmol/l). After exclusion of 17 subjects who did not meet the inclusion criteria, a total of 95 diabetic subjects were randomized into 3 groups; group I received bitter melon 2 g/day (n = 32), group II received bitter melon 4 g/day (n = 33) and group III received glibenclamide 2.5 mg/day (n = 30) for 10 weeks. 5 patients violated the study protocols (2 each in group I and II and 1 in group III) and did not complete the study.

Bitter melon powder was given in the form of capsules (1000 mg) and glibenclamide in the form of tablets. Doses of bitter melon used in this study are based on previously reported improvement of blood glucose levels in clinical trials [22]. Powdered rice (roasted) and lactose were used as placebo for bitter melon and glibenclamide respectively.

### Procedures and measurements

All participating subjects reported fasting in the morning after 12-14 h overnight fast and omission of any morning doses of glucose lowering agents. Venous blood samples were collected without the use of a tourniquet. After screening, the baseline visit (randomization) was scheduled 2 weeks later. FPG and PSA were measured at baseline and then after every week for 10 weeks. HbA1-c, blood lipids and SBP were measured at baseline and at the end point. These parameters were measured with reagents prepared by Boehringer Mannheim (Lewis, UK). Measurement of HbA1c was carried out through Fast ion-exchange resin separation method.

Therapeutic goal was to achieve statistically significant decreases in HbA1-c levels while observing the changes in PSA. Secondary end points include mean changes in FPG, OGTT and blood lipids.

### Source and preparation of bitter melon

Unripe bitter melon fresh fruit obtained from Agriculture farm Sarai Naurang (Lakki Marwat) was washed thoroughly with water, cut open and the seeds removed. The fruits were then processed by modifying the method proposed by Fuangchan et al. [22]. The juice was extracted from the fruit pulps by using electric juicer which was then tested for microbial contamination and/or the presence of heavy metals by the Departments of Basic Medical Sciences and Pharmaceutical Sciences respectively, Gandhara College of Pharmacy, Gandhara University, Peshawar, Pakistan. The non-contaminated juice (460 ml/Kg) was frozen at -20°C and then freeze dried for 72 h. The yield was 14.5 g powder/460 ml of juice. When tested for the active constituent charantin and protein content as described earlier [24, 25], the mean yields were 0.085% and 0.110 mg/g of powder respectively.

### Statistical methods

Results are expressed as means ± SD. Data analysis were carried out using SPSS and a Р ≤ 0.05 was taken as statistically significant. Non-parametric statistical methods were used due to unsymmetrical distribution of most variables. Comparisons among groups were made by differences of mean changes in HbA1-c, FPG, OGTT, PSA, blood lipids, body weight and SBP using Mann-Whitney *U*-test and Krushkal-Wallis one-way ANOVA test.

## Results

Table 1 shows the general characteristics of study groups (after cutting outliers) at baseline. There were no significant differences in the mean values of variables among the groups. Subjects in all the groups were overweight with the males higher in ratio than females. The proportion of subjects who completed the study was 94% each for group I and II and 97% for group III.

### Changes in glycohemoglobin

**Figure 1:**
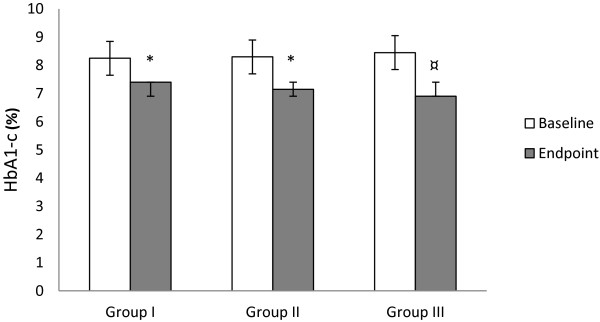
**Effect of bitter melon and glibenclamide on HbA1-c level.** Group I = Bitter melon (2 g/day); Group II = Bitter melon (4 g/day); Group III = Glibenclamide (2.5 mg/day). *p ≤ 0.05 compared to baseline, ^¤^ *p* ≤ 0.01 compared to baseline, ^†^ *p* ≤ 0.05 compared to group III at week 10.

Compared to baseline, we observed significant decreases in the mean levels of HbA1-c at the end point among patients of group I, group II and group III with the later showing greater reduction than the formers (8.25 ± 0.70 vs. 7.40 ± 0.50, p ≤ 0.05; 8.30 ± 0.55 vs. 7.15 ± 0.60, p ≤ 0.02; 8.45 ± 0.60 vs. 6.90 ± 0.75, p < 0.005 respectively). Between groups comparison at the end point revealed no significant difference (Figure 1).

### Changes in plasma sialic acid

**Figure 2:**
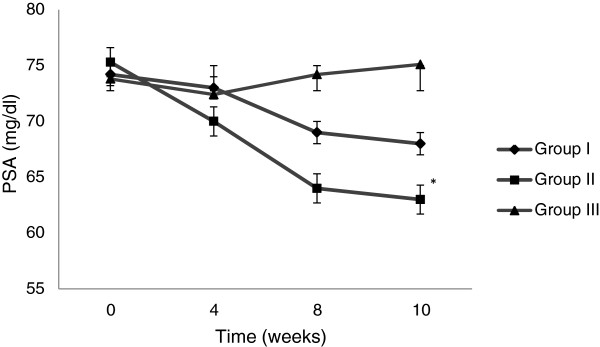
**Changes in PSA overtime.** Group I = Bitter melon (2 g/day); Group II = Bitter melon (4 g/day); Group III = Glibenclamide (2.5 mg/day). *p ≤ 0.05 compared to baseline, ^¤^ *p* ≤ 0.01 compared to baseline, ^†^ *p* ≤ 0.05 compared to group III at week 10.

PSA showed a trend towards decreased level from baseline to endpoint among patients of group I and group II, but the temporal pattern of these reductions was different among the groups. In group I, the level decreased from 74 ± 8.10 mg/dl at baseline to 68 ± 6.70 mg/dl at the endpoint (p < 0.08), whereas in group II, statistically significant decrease occurred from 75.30 ± 8.20 mg/dl at baseline to 63 ± 6.70 mg/dl at the endpoint (p < 0.01). Moreover, the decrease was more rapid in group II compared to group I. In group III, the level slightly deteriorated and rose from 73.90 ± 7.20 mg/dl at baseline to 75.10 ± 5.20 mg/dl at the endpoint (p ≤ 0.11). The mean difference was significant at the endpoint between group II and group III (P ≤ 0.05) (Figure 2).

### Changes in fasting plasma glucose

FPG levels significantly decreased from baseline to endpoint in group I, group II and group III with the group III showing greater reduction (146 ± 13.40 mg/dl vs. 133.70 ± 11.50 mg/dl, p ≤ 0.05; 141.60 ± 15.20 mg/dl vs. 126.40 ± 11.90 mg/dl, p < 0.04; 143.50 ± 18.40 mg/dl vs. 117 ± 10.30 mg/dl, p < 0.003 respectively). The mean difference was significant (*p* ≤ *0.05*) between group I and group III at the endpoint (Table 2).

**Table 2:** **Effect of bitter melon and glibenclamide on clinical/metabolic variables of study groups**

Variables	Baseline	Endpoint (week 10)
FPG (mg/dl)		
Bitter melon 2 g/day (Group I)	146±13.40	133.70±11.50*^†^
Bitter melon 4 g/day (Group II)	141.60±15.20	126.40±11.90*
Glibenclamide 5 mg/day (Group III)	143.50±18.40	117±10.30^¤^
2 h post OGTT plasma glucose (mg/dl)		
Bitter melon 2 g/day (Group I)	241.30±15.60	235±19.50
Bitter melon 4 g/day (Group II)	247±21.90	240.70±25.10
Glibenclamide 5 mg/day (Group III)	255.60±26.30	229.40±20.80*
T C (mg/dl)		
Bitter melon 2 g/day (Group I)	218.25±10.70	214.50±14.50
Bitter melon 4 g/day (Group II)	227.10±15.50	221±11.60
Glibenclamide 5 mg/day (Group III)	220.50±11.10	222±10.80
LDL-c (mg/dl)		
Bitter melon 2 g/day (Group I)	149.70±18.20	146.50±11.50
Bitter melon 4 g/day (Group II)	154±15.50	148.90±16.40
Glibenclamide 5 mg/day (Group III)	146.80±10.30	148.50±12.35
HDL-c (mg/dl)		
Bitter melon 2 g/day (Group I)	48.15±4.90	50±3.50
Bitter melon 4 g/day (Group II)	47.60±5.70	51.20±4.20
Glibenclamide 5 mg/day (Group III)	48.10±5.50	45±4.90
T G (mg/dl)		
Bitter melon 2 g/day (Group I)	163.20±17.50	159.80±16.70
Bitter melon 4 g/day (Group II)	168±13.40	154.20±11.80*
Glibenclamide 5 mg/day (Group III)	165.50±20.50	172.90±18.40
T C/HDL-c ratio		
Bitter melon 2 g/day (Group I)	4.53±1.10	4.29±1.20
Bitter melon 4 g/day (Group II)	4.77±1.60	4.31±1.35
Glibenclamide 5 mg/day (Group III)	4.58±1.15	4.93±1.50
LDL-c/HDL-c ratio		
Bitter melon 2 g/day (Group I)	3.10±1.35	2.93±1.10
Bitter melon 4 g/day (Group II)	3.23±1.52	2.89±1.31
Glibenclamide 5 mg/day (Group III)	3±1.47	3.30±1.20
SBP (mmHg)		
Bitter melon 2 g/day (Group I)	139±8.10	135.50±11.90
Bitter melon 4 g/day (Group II)	144.80±23.70	133.10±14.30*
Glibenclamide 5 mg/day (Group III	136.20±12.24	135.50±10.50
Weight (Kg)		
Bitter melon 2 g/day (Group I)	69.50±10.70	67.65±11.32
Bitter melon 4 g/day (Group II)	73.90±12.20	70.80±10.50
Glibenclamide 5 mg/day (Group III	68.40±8.30	70±11.90

*p≤0.05 compared to baseline; ^¤^ *p*≤0.01 compared to baseline; ^†^ *p*≤0.05 compared to group III at week 10.

### Oral glucose tolerance test and changes in blood glucose levels

The changes observed in 2 hour plasma glucose levels after OGTT were non-significant both in group I and group II (241.30 ± 15.60 vs. 235 ± 19.50, p < 0.16; 247 ± 21.90 vs. 240.70 ± 25.10, p < 0.10 respectively). However, a significant change in 2 hour plasma glucose level after OGTT was observed in group III (255.60 ± 26.30 vs. 229.40 ± 20.80, p < 0.03). Comparison of the mean changes in 2 hour plasma glucose levels between groups revealed no significant difference (Table 2).

### Effect on blood lipids and atherogenic index

The mean changes occurred in blood lipids of groups I, II and III from baseline to endpoint are shown in Table 2. Total cholesterol changed by -3.75 mg/dl, -4.10 mg/dl and +1.50 (p < 0.10, p < 0.06 and p < 0.12 respectively), low density lipoprotein-cholesterol changed by -3.20 mg/dl, -5.10 mg/dl and +1.70 mg/dl (p ≤ 0.06, p < 0.01 and p < 0.12 respectively), high density lipoprotein-cholesterol changed by +1.85 mg/dl, +3.60 mg/dl and -2.10 mg/dl (p < 0.11, p < 0.09 and p < 0.13 respectively), and triglyceride changed by -3.40 mg/dl, -13.80 mg/dl and +7.40 mg/dl (p ≤ 0.07, p ≤ 0.05 and p < 0.08 respectively). Unlike group III, the ratios of TC/HDL and LDL/HDL improved in group I and group II from baseline to endpoint but the mean difference between groups was non-significant (Table 2).

### Effect on systolic blood pressure and body weight

The mean reduction in SBP from baseline to endpoint was non-significant in group I (p ≤ 0.06) but significant in group II (p < 0.05). In group III, there was a slight increase in SBP (p < 0.20). The mean change in body weight from baseline to endpoint among patients of group I, group II and group III was -1.85 Kg (p < 0.09), -3.10 Kg (p < 0.06) and +1.60 Kg (p < 0.25) respectively (Table 2).

During the course of the present study, few gastrointestinal (GI) disturbances occurred among patients receiving bitter melon. These include heartburn and diarrhea (9.3%, 6.2% and 15.15%, 9% in group I and group II respectively). We did not observe GI disturbances among patients receiving glibenclamide, but 6.6% of the patients suffered from skin rashes (Table 3).

**Table 3:** **Unwanted effects of bitter melon and glibenclamide observed during the study period**

	Group I (n= 30)	Group II (n= 31)	Group III (n= 29)
**Gastrointestinal tract**			
Hyperacidity/Heart burn	3 (9.3)	5 (15.1)	1 (3.4)
Anorexia	5 (16.6)	5 (15.1)	3 (10.3)
Nausea	2 (6.6)	3 (9.6)	1 (3.4)
Diarrhoea	1 (3.3)	2 (6.4)	-
Appetite	2 (6.6)	5 (16.1)	3 (10.3)
Abdominal discomfort	1 (3.3)	1 (3.2)	-
**Central Nervous System**			
Headache	4 (13.3)	9 (29)	5 (17.2)
Dizziness	2 (6.6)	1 (3.20	2 (6.8)
**Dermatological**			
Skin rashes	-	2 (6.4)	1 (3.4)

## Discussion

To the best of our knowledge, only few randomized, controlled clinical trials have been conducted to evaluate and compare the hypoglycemic effect of bitter melon fruit pulps with commonly used oral hypoglycemic agents, but most of these studies have conflicting results about the hypoglycemic effect of bitter melon and there is lack of information about its effect on diabetes associated CV risk factors [20, 26]. Results of the present study indicate significant reductions in HbA1-c levels from baseline to endpoint among patients receiving bitter melon (group I, group II) and glibenclamide (group III) with the group III showing greater reduction. At the endpoint, the difference in mean reduction of HbA1-c levels between the groups was non-significant. These results are in agreement with the recent findings by Fuangchan et al. [22] which show a significant decrease in fructosamine level of bitter melon treated type 2 diabetics but in contrast with the previous study reporting a non-significant decline in HbA1-c levels of type 2 diabetics after taking bitter melon extract for 3 months [27]. However, the study has been criticized due to lack of information about the standardization of bitter melon product. Differences in the processing of bitter melon preparation may affect the activity and the hypoglycemic effect of aqueous extract of fruit is reported to be more than the dried fruit powder [21].

A significant decrease in PSA level from baseline to endpoint was observed only in a group receiving bitter melon 4 g/day. Sialic acid is present in glycoconjugates as terminal monosaccharide and is a possible risk factor for CVD in diabetics as well as in general population [28–30]. Its high plasma level reflects the ongoing atherosclerotic process [31]. Earlier, we have shown significant improvement in PSA and cholesterol levels of type 2 diabetics following bitter melon fruit juice and the failure of dried powder of bitter melon 2 g/day (group I) to produce significant reduction in PSA confirms the hypothesis that aqueous extract of the bitter melon fruit is more efficacious than the dried fruit powder [21]. Although, we do not have a solid reason but it might be possible that drying and further processing during powder preparation may cause loss and/or inactivation of some active ingredients notably the polypeptide-p. The group receiving glibenclamide showed a slight increase in the level of PSA from baseline to endpoint. There is a direct relationship between sialic acid and blood lipids and the increase in PSA level may be the result of unfavorable effects of glibenclamide on blood lipids.

The mean changes in 2 hour plasma glucose levels after OGTT were non-significant whereas the changes in FPG levels were significant in group I and II. In group III, the mean changes in 2 hour plasma glucose after OGTT and FPG levels from baseline to endpoint were significant. The previous findings by Srivastava et al. [21] and Fuangchan et al. [22] also showed significant reductions in FPG levels in bitter melon treated subjects. Recently conducted randomized, clinical control trial report no significant changes either in OGTT or FPG levels of type 2 diabetics after taking bitter melon for 4 weeks [22]. However, the use of hot air oven for drying fruit pulp (60°C for 24 hours) may caused denaturation and inactivation of polypeptide-P and other compounds thereby decreasing the hypoglycemic effect of bitter melon [25, 32]. In addition, the lower charantin content/g of the dried fruit pulp in the study by Fuangchan et al. [22] than the present study might be due to the use of heat for drying purposes.

Unlike the group receiving glibenclamide in which the blood lipid levels deteriorated, the groups receiving bitter melon showed favorable changes in the blood lipid levels from baseline to endpoint, but did not reach the statistical significance except the triglyceride level of a group receiving bitter melon 4 g/day (group II). Moreover, the mean TC/HDL ratio was found to be low among patients of group I and group II than group III. TC/HDL ratio is more sensitive and specific index of CV risk than TC alone, specially with values >6 [33]. According to Framingham Heart Study, total cholesterol-to-HDL ratio of 5 signifies for men an average risk of heart disease whereas for women, a ratio of 4.4 signifies an average risk [34]. Treatment with bitter melon 2 g/day and 4 g/day was also associated with decreases in body weight and SBP from baseline to endpoint, but the decrease was significant only in the body weight of a group receiving bitter melon 4 g/day. These wanted effects were not observed with glibenclamide that rather exerted deteriorating effect.

Controversies about the hypoglycemic effects of BM exist due to differences in the processing of its preparation and will continue to exist until a validated standardized method for the preparation of BM formulation is not developed. The present study favors the hypoglycemic effect of bitter melon among type 2 diabetics at the dose of 2 g/day and 4 g/day however, the glycemic control was poor compared to glibenclamide. In contrast, the effect on diabetes associated CV risk factors was more favorable by BM than glibenclamide, suggesting the BM to be less hypoglycemic but more antiatherogenic than glibenclamide.

## Abbreviations

CV: Cardiovascular
FPG: Fasting plasma glucose
F: Female
GI: Gastrointestinal
HDL-c: High density lipoprotein cholesterol
LDL-c: Low density lipoprotein cholesterol
M: Male
OGTT: Oral glucose tolerance test
PSA: Plasma sialic acid
SBP: Systolic blood pressure
TC: Total cholesterol
TG: Triglyceride
WHO: World Health Organization.
